# Protocol for a Multistage Mixed-Methods Evaluation of Multidisciplinary Chronic Kidney Disease Care Quality Following Integration of Virtual and In-Person Care During the COVID-19 Pandemic

**DOI:** 10.1177/20543581221103103

**Published:** 2022-06-01

**Authors:** Micheli Bevilacqua, Helen Chiu, Yuriy Melnyk, Janet Williams, Robin Chohan, Julie Wei, Dominik Stoll, Michele Fryer, Marlee McGuire, Anne Logie, Paul Watson, Adeera Levin

**Affiliations:** 1 Faculty of Medicine, Division of Nephrology, The University of British Columbia, Vancouver, Canada; 2 BC Renal, Provincial Health Services Authority, Vancouver, Canada; 3 Office of Virtual Health, Provincial Health Services Authority, Vancouver, BC, Canada

**Keywords:** chronic kidney disease, multidisciplinary care, virtual care, telehealth, quality of care

## Abstract

**Background:**

Multidisciplinary care of patients with chronic kidney disease (CKD) as it previously existed was predicated on an evidence and experience base of improved patient outcomes within an established and well-described service delivery model. The onset of the COVID-19 pandemic brought with it a departure from this established care delivery model toward integration of virtual care and in-person care.

**Objective:**

To develop an evaluation framework to determine whether this shift in service delivery models has affected quality of multidisciplinary kidney care and/or patient-clinician interactions and relationships.

**Design:**

A sequential multiphase, mixed-methods evaluation.

**Setting:**

All 15 British Columbia (BC) multidisciplinary kidney care clinics (KCCs).

**Participants:**

All patients and all clinicians in all KCCs across BC will be invited to participate in the planned evaluation.

**Measurements:**

Qualitative and quantitative feedback from patients and families living with CKD and KCC clinicians.

**Methods:**

The planned multiphase evaluation of virtual care integration in KCCs will be conducted across all 15 KCCs in the province of BC, Canada. The following phases are proposed: (1) review of current virtual care integration and practices, (2) assessment of patient and clinician experiences and perspectives via semi-structured interviews, (3) validation of those patient and clinician perspectives via survey of a larger sample, (4) compilation and analysis of all phases to provide informed recommendations for patient and visit format selection in a mixed in-person and virtual multidisciplinary clinic setting.

**Limitations:**

This work will not capture any information about the relationship between differences in virtual usage parameters and clinical outcomes or financial implications.

**Conclusions:**

There is no existing framework for either evaluation of multidisciplinary CKD care quality in a virtual setting or evaluation of care quality following a substantial change in service delivery models. The proposed evaluation protocol will enable better understanding of the nuances in kidney care delivery in this new format and inform how best to optimize the integration of virtual and pre-existing formats into kidney clinic care delivery beyond the pandemic. Beyond the current evaluation, this protocol may be of use for other jurisdictions to evaluate their own local instances of virtual care implementation and integration. The model may be adapted to evaluate quality of multidisciplinary kidney care delivery following other changes to clinic service delivery models.

## Abrégé

**Contexte:**

Les soins multidisciplinaires prodigués aux patients atteints d’insuffisance rénale chronique (IRC), tels qu’ils existaient auparavant, étaient fondés sur une base de preuves et d’expérience de l’amélioration des résultats pour les patients dans le cadre d’un modèle de prestation de services établi et bien décrit. Le début de la pandémie COVID-19 a créé une fracture par rapport à ce modèle établi de prestation de soins, à la faveur d’une intégration des soins virtuels et des soins en personne.

**Objectif:**

Élaborer un cadre d’évaluation permettant de déterminer si ce changement dans les modèles de prestation de services a eu une incidence sur la qualité des soins multidisciplinaires en santé rénale et/ou sur les interactions et les relations entre le patient et le clinicien.

**Type d’étude:**

Une évaluation multiphase séquentielle à méthodes mixtes

**Cadre:**

Les 15 cliniques multidisciplinaires de soins rénaux (CMSR) de la C.-B.

**Participants:**

Tous les patients et cliniciens de toutes les CMSR de la Colombie-Britannique seront invités à participer à l’évaluation

**Mesures:**

Les rétroactions qualitative et quantitative des patients atteints d’IRC et de leurs familles, ainsi que celles des cliniciens des CMSR.

**Méthodologie:**

L’évaluation multiphase prévue de l’intégration des soins virtuels dans les soins prodigués dans les CMSR sera menée dans les 15 CMSR de la Colombie-Britannique (C.-B.), au Canada. Les phases suivantes sont proposées: (1) examen de l’intégration et des pratiques actuelles en matière de soins virtuels, (2) évaluation des expériences et des perspectives des patients et des cliniciens par le biais d’entrevues semi-structurées, (3) validation de ces mêmes perspectives par le biais d’un sondage sur un échantillon plus large, (4) compilation et analyze des données recueillies lors de toutes les phases afin de fournir des recommandations éclairées pour le choix des patients et du format de la visite dans un contexte de clinique multidisciplinaire intégrant les consultations en personne et virtuelles.

**Limites:**

Ces travaux ne permettront pas de recueillir des informations sur la relation entre les différences dans les paramètres d’utilization virtuelle et les résultats cliniques ou les implications financières.

**Conclusion:**

En ce moment, il n’existe pas de cadre pour évaluer la qualité des soins multidisciplinaires en IRC dans un cadre virtuel ni pour évaluer la qualité des soins après un changement important dans les modèles de prestation de services. Le protocole d’évaluation proposé permettra de mieux comprendre les nuances dans la prestation des soins rénaux dans ce nouveau format et d’indiquer la meilleure façon d’optimiser l’intégration des formats virtuels et préexistants dans la prestation des soins en clinique rénale au-delà de la pandémie. Au-delà de l’évaluation actuelle, ce protocole pourra être utilisé par d’autres provinces pour évaluer leurs propres instances locales de mise en œuvre et d’intégration des soins virtuels. Le modèle peut être adapté pour évaluer la qualité de la prestation de soins multidisciplinaires aux reins à la suite d’autres changements apportés aux modèles de prestation de services en clinique.

## Introduction

Non-dialysis chronic kidney disease (CKD) care is complex. Many jurisdictions have addressed this complexity through multidisciplinary clinics which are founded in an existing evidence and experience base.^1,2^ The onset of the COVID-19 pandemic brought a dramatic change and departure from the traditional model of multidisciplinary care delivery including a rapid shift to mostly virtual care delivery early in the pandemic, with strategies to integrate virtual and in-person care developed and integrated thereafter.^3^ Virtual care in this setting refers to any situation where a patient is not physically present in the clinic space, including encounters by phone, video-enabled platforms, or some combination thereof.

Importantly, this substantial shift in care delivery was not the result of a planned change or program of work focused on a desired endpoint but rather a response to the emergent reality of a public health crisis. Much of the proven success and the key activities of multidisciplinary clinics are predicated on a robust and longitudinal relationship between the care team and the patient. For example, in British Columbia (BC), kidney care clinic (KCC) teams have traditionally met with patients, and where appropriate family/support people, in a physical clinic setting which allows not just for physical contact and examination, but also enables the nuanced conversations and decision-making involved in kidney care.^1,2^ The ability of the team to understand each patient’s unique values and goals of care is integral in optimizing patient-centered CKD care and is dependent on quality interactions between patients and care providers.^1,2,4^ With the rapid shift to largely virtual models implemented in a variety of methods in response to the COVID-19 pandemic, it is unclear if the existing model of multidisciplinary care or the foundational high-quality patient and provider interactions were preserved.

Reported experiences with virtual care implementation in kidney care and other care settings largely describe feasibility of implementing virtual solutions,^5-9^ access, and/or user acceptability of virtual tools.^8-11^ There are limited data on the quality of these care interactions across the spectrum of different visit modalities and types of clinical interactions involved in multidisciplinary CKD care. In addition, these evaluations largely focus on the implementation of a specified virtual clinic model in comparison with traditional in-person clinic services, but not the integration of the two, which was the real-world response to the COVID-19 pandemic.^3,12^ This is important as inherent in any hybrid model combining in-person and virtual care delivery, is the need to determine which visit modality to use for any given interaction. Similarly, existing methods to evaluate quality of multidisciplinary CKD care do not capture this unique situation either. Existing quality metrics for CKD care are mainly at the clinic level and focused on adherence to guidelines and care pathways,^13^ rather than evaluating methods and quality of individual clinical encounters. The patient-reported experience measures (PREMs) that do assess the quality of these encounters^14,15^ including the tool used for evaluation of patient experience within BC^16^ were not designed for use in virtual settings, and do not include considerations specific to virtual care delivery.

The need to alter service delivery rapidly during the pandemic while continuing to deliver longitudinal care for chronic complex diseases such as CKD has highlighted a limitation in evaluating KCC service delivery. There is a paucity of information on how to effectively integrate new and traditional care models, and more broadly, how to evaluate the quality and value provided by multidisciplinary kidney care delivery models and interactions either in steady-state or when those delivery models are changing. Because of these limitations, and out of a desire to inform optimal integration of traditional and virtual care models, we report here a protocol to evaluate the implementation, delivery, and patient and clinician experiences associated with the combinations of visit types and modalities that were experienced in the wake of the COVID-19 pandemic in KCCs across BC, Canada. This structured evaluation approach will be necessary to inform the goal of enhancing KCC offerings by determining optimal method(s) to integrate and individualize virtual and in-person modes of care delivery currently and beyond the pandemic.

## Methods

### Study Setting

All phases of this evaluation study were planned and are to be conducted in BC, Canada. BC Renal (BCR) is the provincial network responsible for coordinating all kidney care across this large and diverse province, including extensive experience with and a robust provincial structure for KCC care delivery.^4^ Through BCR, CKD care is funded and coordinated centrally but delivered locally through KCCs, such that provincial committees with diverse representation set direction and guidelines for the KCCs, but clinic operations are out of the purview of BCR and directed locally by the health authorities in which those clinics exist.^4^ In BC, there are currently 15 KCCs across 5 geographic health authorities, and 1 provincial pediatric program which together delivery CKD care for more than 12,000 CKD patients. These KCCs represent a diverse array of sizes, geography, populations served, staffing, and local resources. Despite this variation, prior to the pandemic, KCC care in BC was fairly standardized according to established clinic best practices.^4^ Following the onset of the pandemic, each KCC responded with their own approach to implementing virtual procedures and solutions based on their unique needs, resources, local/health authority regulations, support, and platform availability.

### Timelines, Dedicated Evaluation Working Group Formation, Composition, and Guiding Principles

Following the shift to virtual care in March 2020, the BCR committee responsible for KCC care coordination (BC Renal Kidney Care Clinic Committee; KCC Committee) met with key stakeholders and agreement was reached to form a Virtual Care Clinical Working Group to enable sharing of experiences and best practices during this transition to include virtual care. As it became clear that this transition was not a short-term or time-limited change to virtual care, BCR recognized the need for a more thorough and structured evaluation of virtual care to better inform its usage in both the short and longer term. To enable this, BCR provided resourcing and infrastructure support to enable this and in January 2021, a Virtual Care Evaluation Working Group was formed.

The Evaluation Working group consists of 2 kidney health professionals, a project manager, a quality improvement specialist, 2 representatives from BC’s Provincial Health Services Authority (PHSA) Office of Virtual Health (OVH), and 2 patient partners with lived experience of CKD care in BC KCCs. In addition to these core working group members, the working group engages input from BCR members with additional expertise, for example, for specific methodological, organizational, or analytic support. Members of the Working Group are responsible for the overall evaluation design, preparation of evaluation components, progress monitoring and execution, administration, analysis, and reporting of all surveys and interviews. The working group reports to the BCR KCC Committee which provides oversight and will ultimately be responsible for developing best practice guidance and integrating recommendations from the evaluation reports into ongoing practice of the KCCs across BC.

A guiding principle of the Evaluation Working Group is to use existing evaluation frameworks where possible to inform this tailored evaluation approach. Two frameworks used heavily are BCR’s internal evaluation framework^17^ and the BC Health Quality Matrix.^18^ The BC Health Quality Matrix is a tool developed by the BC Patient Safety and Quality Council and has been widely used across diverse care settings in BC to evaluate care quality by defining and evaluating discrete domains that contribute to quality of care (Table 1). These frameworks serve as a foundation to which feedback from working group members and key stakeholders is integrated to develop the evaluation protocol below.

**Table 1.** Components of Quality Care Delivery and Definitions; Adapted From BC Health Quality Matrix.^18^

Dimension of quality care delivery	Definition
Respect	Honoring a person’s choices, needs, and values
Safety	Avoiding harm and fostering security
Accessibility	Ease with which health and wellness services are reached
Appropriateness	Care that is specific to a person’s or community’s context
Effectiveness	Care that is known to achieve intended outcomes
Equity	Fair distribution of services and benefits according to population need
Efficiency	Optimal and sustainable use of resources to yield maximum value

*Note.* BC = British Columbia.

### Overview of Study Design

**Figure 1.**
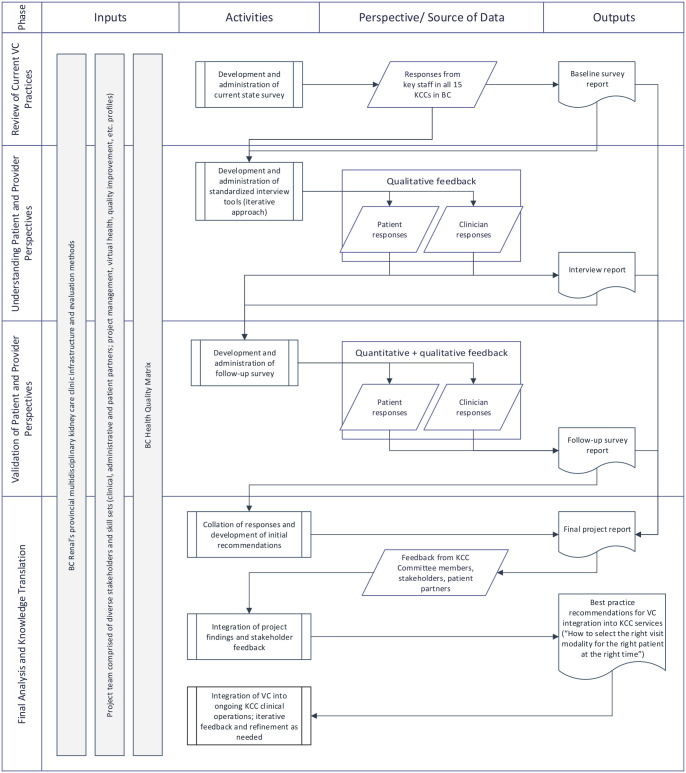
Evaluation design overview. *Note.* BC = British Columbia; KCC = kidney care clinic; VC = virtual care.

An overview of the evaluation study flow is illustrated in Figure 1. The proposed study involves a stepwise mixed-methods approach.

#### Phase 1: Review of current virtual care integration and practices across BC KCCs

**Figure 2.**
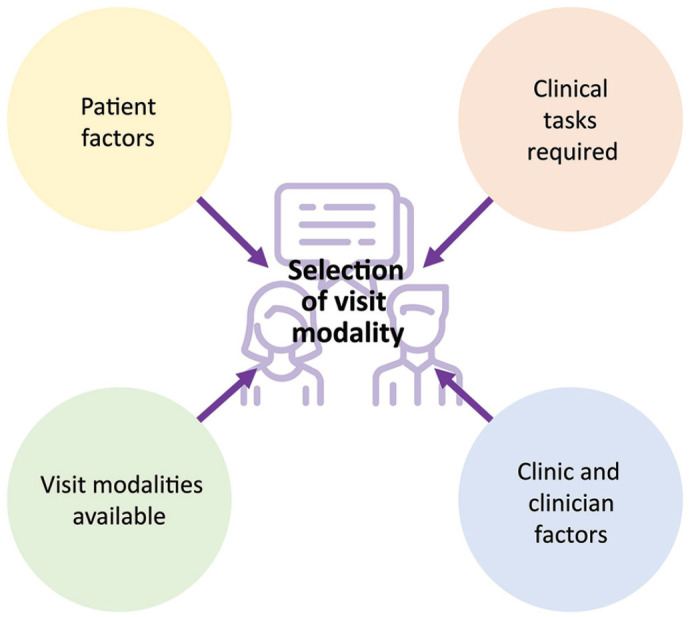
Factors influencing selection of visit modalities in a mixed in-person and virtual multidisciplinary kidney clinic.

To assess the current state of integration of virtual care with previous clinic modalities in KCCs, we will first examine the baseline characteristics of virtual care implementation across the 15 KCCs in BC. A series of survey questions related to the adoption of various visit modalities in their current practices as well as operational and workflow for virtual health will be developed. In addition to visit formats of virtual or in-person, questions will be asked about specific visit purposes as this in combination with patient and team-specific factors may influence the choice of visit modalities; in KCCs, visit purposes can include orientation visit, routine team-based visit, encounters between clinic visits, and/or education-focused visits such as those for transplant or dialysis modalities (Figure 2). This first phase will be planned as an online survey, and clinical operations representatives from each of the 15 KCCs will be invited to participate in the survey within a 3-week period. The survey results will be analyzed with a report constructed to describe the current landscape of virtual care integration in BC KCCs. This information will be used to inform the development of Phase 2.

#### Phase 2: Understanding patient and clinician perspectives of virtual care usage and integration

Semi-structured interviews will be used to understand key patient and provider perspectives of their experiences with different formats and considerations around the optimal use and integration of in-person and different virtual visit modalities. The interview questions will be designed based on the findings from Phase 1 to ensure relevance to the current landscape of KCC services in BC. These questions will be tested and fine-tuned in the pilot interviews of each participant groups. As there have been several waves of the pandemic with resultant ebbs and flows in restrictions and access that patients may have experienced, the questioning will be posed to elicit respondents’ experiences through the totality of changes since the pandemic, rather than focusing on the specific situation at any particular snapshot in time.

Patients and KCC clinicians who have had in-person and virtual visits at a KCC in BC during the pandemic will be invited to participate in the interviews *via open invitations* which will be shared. Interview participants may choose to participate via phone or video conference at a time convenient to them. Patient interviews will be offered in English and other languages commonly used by patients receiving care in BC. All interviews will be recorded and transcribed verbatim.

Interview transcripts will be coded and analyzed using NVivo (QSR International). Data will be sorted into key themes and sub-themes of key considerations for determining use of in-person versus virtual options to provide optimal kidney care. A specific recruitment target will not be set in advance; while recruitment is underway, the qualitative data will be analyzed simultaneously to monitor for diversity in participant demographic (eg, representation from all regions, rural vs urban settings, professional disciplines) and thematic saturation,^19^ and recruitment will continue until such saturation is reached. The 7 dimensions of quality in the BC Health Quality Matrix (Table 1^18^) will be used as reference. Furthermore, the evaluation working group members will adopt a thematic process that includes data condensation, displaying, and conclusion drawing for the qualitative analysis.^20^ Key themes drawn from the data collected in this phase will be validated in Phase 3.

#### Phase 3: Validation of patient and clinician perspectives

A set of surveys will be developed based on the interview findings from Phase 2 for patients and clinicians with questions designed to validate and rate the importance of considerations for leveraging the use of in-person versus virtual options across the spectrum of KCC care. In addition to validating the themes observed in phase 2, the survey will also examine if considerations and preferences vary based on different patient characteristics such as basic demographics, remote versus urban locations, cultural and language background, and socioeconomic status. The survey will be hosted online (Research Electronic Data Capture [REDCap]), and the same recruitment strategies as in Phase 2 will be used. As with the patient interviews, the patient survey will also be offered in multiple languages. The survey will be broadly advertised through the KCCs such that all patients receiving care at a BC KCC and all KCC clinicians will be eligible to participate in the survey over a 3-week period. No specific recruitment target will be set for the survey, but with more than 12,000 KCC patients, even in the potential situation of a low response rate, we anticipate a sufficient number of respondents for analysis. Descriptive statistics will be used to analyze the survey results by participant group. The survey results along with findings from Phases 1 and 2 will be integrated into a final report and inform the development of practice recommendations in Phase 4.

#### Phase 4: Development and integration of practice guides into ongoing KCC care delivery

A final report will synthesize findings from the previous phases. A key component will be a set of actionable recommendations for patient and visit format selection with the goal of providing clinicians with practical tools to discern “the right visit modality for the right patient at the right time.” The BCR KCC Committee will use these recommendations to develop best practice guidance and tool(s) for integration of virtual and in-person visits into ongoing KCC service delivery during and beyond the pandemic. We anticipate that this guidance will be in the form of key considerations that enable KCC staff to engage in informed, shared decision-making to identify the best visit type(s) for each patient encounter. The results of the planned evaluation will inform this process by validating (1) key information for staff to elicit about patients’ individual circumstances and (2) an enhanced understanding of what visit types do and do not work well for certain KCC tasks. Quality indicators and metrics will also be included in the recommendations to enable ongoing progress monitoring, evaluation, and quality improvement by the KCC Committee.

## Discussion

Multidisciplinary CKD care has traditionally been delivered and evaluated within the context of a model of team-based interactions, provided at regular intervals, for the most part face-to-face.^1^ As the pandemic continued, it became clear that the likely and preferred model for ongoing kidney care will be an evolving hybrid of in-person and virtual care.^3,12^ In some ways, this hybrid model is even more challenging than implementing either visit modality in isolation as the composite of the two necessitates a framework for clinicians to choose from options for each individual patient encounter. The rapid nature of this change in service delivery meant that shift to virtual care was implemented before these methods could be studied or guidance developed to enable clinicians to navigate this new care landscape. To better understand the effect of these changes in care delivery on patients and care providers, we have planned a structured, stepwise mixed-methods evaluation. We will evaluate the implementation, delivery, and patient and provider experiences associated with the combination of in-person and virtual care currently provided in BC KCCs with the goal of using these experiences to inform optimal method(s) to combine and individualize these care methods now and beyond the pandemic.

A new evaluation approach is required as no validated tools exist to assess quality of KCC interactions in a hybrid physical and virtual model, or alternatively to evaluate multidisciplinary care after a disruptive change to existing care models. The approach we have developed addresses this challenge by obtaining patient and provider feedback spanning the spectrum of different visit modalities, variety of clinical interactions, clinic resources, and patient needs encountered in multidisciplinary kidney care. This results in a complex matrix of parameters to evaluate (Figure 2), but this complexity is necessary as it reflects the real-world complexity of multidisciplinary CKD care delivery.^1,2^ There likely will not be a single solution but an approach for clinicians to decide between the variety of available solutions depending on patient needs and capacity, clinical situations, logistical factors, and IT support among other potential considerations.^3,12^

With variability inherent in emergent virtual care implementation, local clinic cultures, and makeup of populations served, it will be worthwhile for other jurisdictions to conduct a similar evaluation of their own local instances of virtual or hybrid KCC care. Although this protocol is designed to address the immediate clinical and evaluation need in response to the pandemic, this protocol may be adaptable and useful in the event of future changes or departures from established service delivery models and may even be adaptable to other multidisciplinary chronic disease care settings.

The development and delivery of this evaluation approach is enabled by several factors within BC. Foremost is an established provincial network of kidney care delivery which includes the clinical expertise, and operational and administrative support necessary to coordinate large programs of work such as a provincial scope evaluation of care delivery across diverse settings. While the variation of virtual care practice across BC can be a challenge for standardization, this provincial network facilitates coordination and shared learning among all KCCs in BC. In addition, the existing BCR network allows for ready engagement of a range of KCC subject matter experts, collaboration with virtual care subject matter experts, and most importantly, an existing framework to recruit and engage patient partners.

Some limitations to this protocol exist. This work will not capture any information about the relationship between the changes in KCC service delivery and clinical outcomes or financial implications; these are beyond the current scope of work but remain an important topic for future study. Similarly, physician and clinic remuneration for in-person versus virtual care is another important consideration that affects visit modality selection, but it is out of the direct purview of KCC clinicians and thus has been kept out of scope of the planned evaluation. The evaluation has been structured to prioritize identification of the most clinically appropriate visit method(s) for any given situation, and the practical considerations of funding and how remuneration influences decision making can be further examined thereafter. Another potential limitation is that despite our best efforts, this approach may not be able to completely examine the perspectives of difficult to reach populations due to geographical, language, cultural, and/or socioeconomically distinct factors. To help mitigate this limitation, the representativeness of our data will be closely monitored for any missing perspectives in Phases 2 and 3 and in that way inform areas for more focused and dedicated follow-up examination.

## Conclusion

In conclusion, this proposed evaluation protocol will provide robust and actionable evidence from both the patient and clinician perspectives which will be essential to inform how best to leverage available visit modalities for multidisciplinary kidney care now and beyond the pandemic. This protocol may also be of use and adaptable for other jurisdictions to evaluate and optimize their unique care landscapes following the pandemic and more generally may be adaptable as a model for evaluating multidisciplinary chronic disease care following a disruptive change to service delivery models.
